# Chloroplast Genomes for Five *Skeletonema* Species: Comparative and Phylogenetic Analysis

**DOI:** 10.3389/fpls.2021.774617

**Published:** 2021-12-13

**Authors:** Shuya Liu, Qing Xu, Kuiyan Liu, Yongfang Zhao, Nansheng Chen

**Affiliations:** ^1^CAS Key Laboratory of Marine Ecology and Environmental Sciences, Institute of Oceanology, Chinese Academy of Sciences, Qingdao, China; ^2^Functional Laboratory of Marine Ecology and Environmental Science, Qingdao National Laboratory for Marine Science and Technology, Qingdao, China; ^3^Center for Ocean Mega-Science, Chinese Academy of Sciences, Qingdao, China; ^4^College of Life Science and Technology, Huazhong Agricultural University, Wuhan, China; ^5^College of Marine Science, University of Chinese Academy of Sciences, Beijing, China; ^6^Jiaozhou Bay National Marine Ecosystem Research Station, Institute of Oceanology, Chinese Academy of Sciences, Qingdao, China; ^7^Department of Molecular Biology and Biochemistry, Simon Fraser University, Burnaby, BC, Canada

**Keywords:** *Skeletonema* species, chloroplast genomes, comparative genomics, divergence time, phylogenetic analysis, PETF

## Abstract

*Skeletonema* species are cosmopolitan coastal diatoms that exhibit important roles in ecological system. The chloroplast genomes (cpDNAs) have been proven to be important in the study of molecular evolution and genetic diversity. However, cpDNA of only a single *Skeletonema* species (*S. pseudocostatum*) has been constructed, hindering in-depth investigation on *Skeletonema* species. In this study, complete cpDNAs of five *Skeletonema* species were constructed with cpDNAs of four species *S. marinoi*, *S. tropicum*, *S. costatum*, and *S. grevillea* constructed for the first time. These cpDNAs had similar sizes and same numbers of genes. These cpDNAs were highly syntenic with no substantial expansions, contractions, or inversions. Interestingly, two copies of *petF*, which encodes ferredoxin with critical role in iron dependency, were found in all five *Skeletonema* species, with one copy in the cpDNA and another copy in the nuclear genome of each species. Selection analysis revealed that all PCGs of cpDNAs were undergoing purifying selection. Despite the high conservation of these cpDNAs, nine genomic regions with high sequence divergence were identified, which illustrated substantial variations that could be used as markers for phylogenetic inference and for tracking *Skeletonema* species in the field. Additionally, the numbers of simple sequence repeats varied among different cpDNAs, which were useful for detecting genetic polymorphisms. The divergence times estimated using PCGs of cpDNAs revealed that most of these species were established within ∼33 Mya, consistent with that estimated using mtDNAs. Overall, the current study deepened our understanding about the molecular evolution of *Skeletonema* cpDNAs.

## Introduction

The chloroplasts are organelles for photosynthesis and other biochemical pathways in plants and algae (Halliwell, 1984). Chloroplast genomes (cpDNAs) represent maternal inheritance. A typical cpDNA has a highly conserved circular DNA arrangement with a quadripartite structure with a pair of inverted repeats (IRs: IRA and IRB), which are separated from the large-single copy (LSC) region and the small-single copy (SSC) region (Sabir et al., 2014; Liu and Pang, 2016; Yu et al., 2018; Abdullah et al., 2021; Kim et al., 2021). Research on cpDNAs complements researches on mitochondrial genomes (mtDNAs), which also present uniparental inheritance, and the nuclear genomes (Gould et al., 2008; Garg and Gould, 2016; Xu et al., 2017). Complete cpDNA sequences have been widely accepted as an informative and valuable source for understanding evolutionary biology and ecological applications (Hamsher et al., 2019; Song et al., 2020).

As one of the most diverse and ecologically important groups in phytoplankton, diatoms (Bacillariophyta) contribute about one-fifth of the total photosynthesis on earth (Armbrust, 2009; Malviya et al., 2016). The plastids of Bacillariophyta were derived by a second endosymbiosis in which red algae were taken up by a eukaryotic heterotroph (Armbrust et al., 2004; Armbrust, 2009). With time, genes from the red algae were transferred to the host nuclear genomes and many photosynthetic proteins were now encoded in the nuclear genomes, synthesized in the cytoplasm and imported into the chloroplast (Gould et al., 2008; Garg and Gould, 2016). Such endosymbiotic gene transfers (EGTs) may be critical in evolution. For example, the transfer of the *petF* gene, which encodes for ferredoxin with critical role in iron dependency was found to be transferred from the cpDNA to the nuclear genome in the diatom *Thalassiosira oceanica* (Lommer et al., 2010; Roy et al., 2020). This transfer may be critical for this diatom species to adapt to the low-iron ocean regions (Strzepek and Harrison, 2004).

The phylum Bacillariophyta is estimated to have 12,000 - 30,000 species (Malviya et al., 2016). In contrast, cpDNAs of only 55 Bacillariophyta species have been constructed, hindering further research on the evolution of Bacillariophyta species. Thus, construction and analysis of more Bacillariophyta cpDNAs are urgently needed for understanding their evolutionary history.

The genus *Skeletonema*, which belongs to Skeletonemataceae family, Thalassiosirales order, Mediophyceae class in Bacillariophyta, consisted of 21 taxonomically accepted species (Sarno et al., 2005, 2007; Guiry and Guiry, 2021). *Skeletonema* species have a cosmopolitan distribution and are usually dominant phytoplankton in the coastal seawaters and estuaries (Kooistra et al., 2008; Liu et al., 2020; Cui et al., 2021). As an important primary producer, *Skeletonema* species provide food source for the higher trophic levels (Kooistra et al., 2008). Many *Skeletonema* species, including *S. marinoi*, *S. costatum*, and *S. pseudocostatum*, can form harmful algae blooms (HABs) with negative impact on environments (Godhe et al., 2006; Shuya and Nansheng, 2021). For example, high concentrations of *Skeletonema* species utilize nutrients necessary for other species, and also could cause hypoxia or anoxia (Mohamed, 2018). For their easy survival, fast growth, and absence of toxins, *Skeletonema* species are often used as food source for aquaculture (Hemaiswarya et al., 2011). Because of high ecological relevance, *Skeletonema* species are also used as the model phytoplankton species for genetic diversity and physiological adaptation studies (Johansson et al., 2019). For example, Haernstroem et al. (2011) used *S. marinoi* as a model organism, and studied the genetic structure and diversity of *S. marinoi* in sediment core accumulated over a 100 years. However, genomic resources of *Skeletonema* species are limited. For example, cpDNA of only a single *Skeletonema* species, *S. pseudocostatum*, has been constructed (Hamedi et al., 2019).

The aims of our study are: (1) to provide complete cpDNA sequences in *Skeletonema* genus for further comparative analysis; (2) to find suitable molecular markers to distinguish *Skeletonema* species in species level for ecological application; (3) to understand the genetic and evolutionary characteristics in cpDNAs of *Skeletonema* species. In this study, we successfully constructed complete cpDNAs of five *Skeletonema* species (total six *Skeletonema* strains) and analyzed their genomic structures, phylogenetic relationships, nucleotide substitutions, simple sequence repeats (SSRs), and divergence times, as well as the duplicative transfer of *petF* gene to the nucleus.

## Materials and Methods

### Strain Isolation, DNA Sequencing, cpDNA Construction and Annotation

In this study six *Skeletonema* strains were analyzed, and they were isolated from the coastal waters of China including the Bohai Sea (CNS00100), the Jiaozhou Bay (CNS00166), the Yellow Sea (CNS00243), the Changjiang Estuary (CNS00303), the Beibu Gulf (CNS00342), and the South China Sea (CNS00438), respectively, using single-cell capillary method. They were cultured in the L1 medium (Guillard and Hargraves, 1993). The DNA extraction and sequencing were same as described previously (Wang et al., 2021).

The complete cpDNAs were assembled using GetOrganelle (Jin et al., 2020) using MK372941 (*S. pseudocostatum*) as a reference. The cpDNAs were then validated by aligning sequencing reads against the cpDNAs using BWA (Li and Durbin, 2009) and SAMtools (Li et al., 2009), and the alignments were inspected using IGV (Thorvaldsdottir et al., 2013). After validation, the cpDNAs were annotated using MFannot^1^ with genetic code of 11 Bacterial, Archaeal and Plant Plastid, and were then validated using ORF Finder^2^. To compare with published cpDNAs accurately, we have also inspected and re-annotated the 55 cpDNAs in Bacillariophyta from NCBI. The physical maps of the circular cpDNAs of six *Skeletonema* species were generated with Organellar Genome DRAW (OGDRAW) (Greiner et al., 2019). The quadripartite structures of *Skeletonema* cpDNAs were analyzed using Annotation of Organellar Genomes (GeSeq) (Tillich et al., 2017). The AT-skew and GC-skew were calculated according to the formulae: AT-skew = (A − T)/(A + T) and GC-skew = (G − C)/(G + C).

### Synteny Analysis and Sequence Divergence Analysis

Synteny analysis of six cpDNAs was carried out using Mauve (Darling et al., 2010). Gene arrangements of IR regions were plotted using the R package gggenes (David, 2020). Alignments of six *Skeletonema* cpDNA sequences were visualized using mVISTA in the Shuffle-LAGAN mode (Frazer et al., 2004) with the *S. marinoi* cpDNA (MW679506) as a reference sequence. Nucleotide diversity analysis of cpDNAs was conducted using the DnaSP (Rozas et al., 2017) by sliding window (step size = 200 bp, window length = 600 bp).

Based on the results of nucleotide diversity, nine regions with high sequence divergence were identified. The primers of these regions were designed using the Primer Premier 5.0 (Supplementary Table 1) (Singh et al., 1998). PCRs were performed in volumes of 50 μL containing 2 μL diluted template DNA (about 50 ng), 1 μL forward primer (10 μmol/L), 1 μL reverse primer (10 μmol/L), 25 μL mix (Tiangen, China) and 21 μL ddH_2_O. The reactions were denatured at 94°C for 4 min. Then, the reactions were run for 32 cycles at 94°C for 1 min, 52°C for 1 min 50 s, and 72°C for 2 min and a final extension at 72°C for 10 min. These PCR products were run on 1% agarose gels for checking amplicon lengths.

### Phylogenetic Analysis

The phylogenetic trees of the nine regions were generated using the Maximum Likelihood (ML) method with 1000 bootstrap replicates in megaX (Kumar et al., 2018). The best-fit models were Tamura 3-parameter (T92 + G) for all regions except for CpVIII with General Time Reversible (GTR + G) model, which were obtained by Models.

Phylogenetic relationships within the 61 Bacillariophyta cpDNAs (six cpDNAs were constructed in this study and 55 cpDNAs were downloaded from NCBI) and one outgroup (*Triparma laevis*) were analyzed based on the 86 shared protein-coding genes (PCGs) among them. Amino acid sequence of 86 genes, including *atpA, atpB, atpD, atpE, atpF, atpG, atpI, ccs1, ccsA, chlI, clpC, dnaB, ftsH, groEL, petA, petB, petD, petG, petL, petM, petN, psaA, psaB, psaD, psaF, psaJ, psbB, psbC, psbD, psbE, psbF, psbH, psbI, psbJ, psbK, psbL, psbN, psbT, psbV, psbX, psbY, rbcL, rbcS, rpl11, rpl12, rpl13, rpl14, rpl16, rpl18, rpl19, rpl1, rpl20, rpl23, rpl24, rpl29, rpl2, rpl31, rpl34, rpl35, rpl3, rpl4, rpl5, rpl6, rpoA, rpoB, rpoC1, rpoC2, rps10, rps11, rps12, rps13, rps14, rps16, rps17, rps18, rps20, rps2, rps3, rps4, rps5, rps7, rps9, secA, secY, tatC, and ycf3*, were individually aligned by MAFFT (Katoh and Standley, 2013), trimmed by trimAl (Capella-Gutierrez et al., 2009), and concatenated by Phyutility (Smith and Dunn, 2008). The model for the phylogenetic tree was LG + F + R10, which was obtained by ModelFinder (Kalyaanamoorthy et al., 2017). Phylogenetic tree was constructed by IQ-TREE (Trifinopoulos et al., 2016) with SH-aLRT support (%)/aBayes support/ultrafast bootstrap support (%) and displayed by FigTree^3^.

### Simple Sequence Repeat Analysis and Selection Analysis

MISA (Beier et al., 2017) was used to search for SSRs in the cpDNA of *Skeletonema* species. The minimum number repeats were 10, 5, 4, 3, 3, and 3 for mono-, di-, tri-, tetra-, penta-, and hexa-nucleotides, respectively.

**FIGURE 1:**
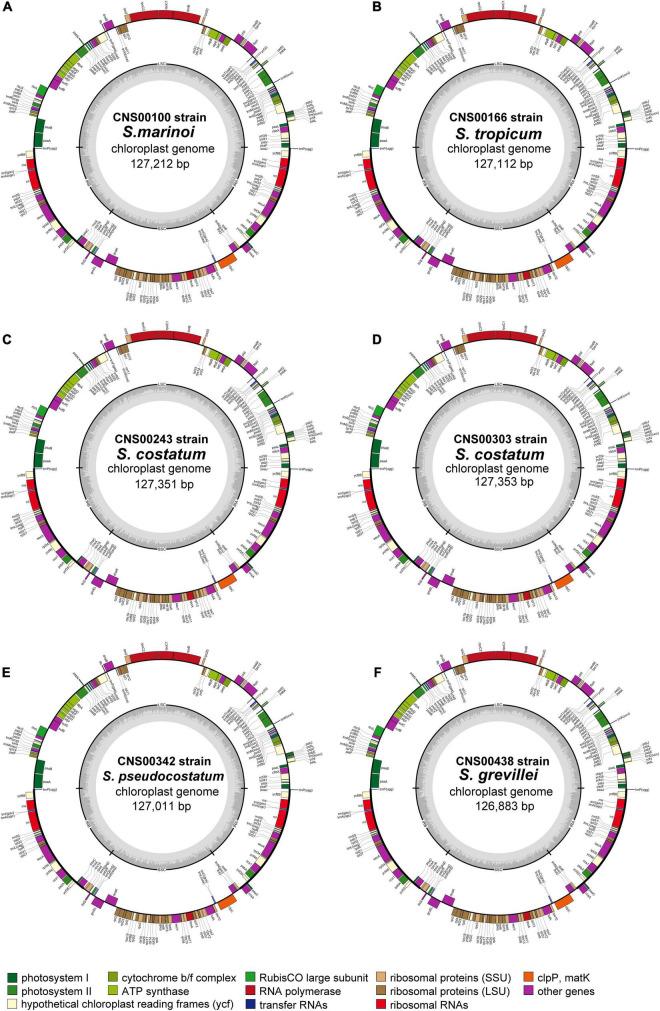
Chloroplast genomes of six *Skeletonema* strains **(A–F)** constructed in this study. Genes inside and outside of the circle are transcribed clockwise and counterclockwise, respectively. Genes belonging to different functional groups are shown in different colors. The LSC and SSC regions are separated by the inverted repeats (IRA and IRB). Dark and light gray colors in the inner circle correspond to the GC and AT content, respectively.

To estimate selection pressure, the rates of non-synonymous (Ka) and synonymous (Ks) substitutions were analyzed by KaKs_calculator2 (Wang et al., 2010). Ka/Ks values > 1 indicated positive selection; and Ka/Ks < 1 indicated purifying selection. Each PCG was calculated pairwise between two *Skeletonema* species, respectively. The results divided into different functional groups (same to Figure 1 generated by OGDRAW) were visualized by the box charts using the R package ggpubr (Kassambara, 2020).

### Divergence Time Estimations

Molecular dating analysis was conducted by 96 cpDNA PCGs with DNA sequences in the Class Mediophyceae. The 96 PCGs were *atpA, atpB, atpD, atpE, atpF, atpG, atpH, atpI, ccs1, ccsA, chlI, clpC, dnaB, dnaK, ftsH, groEL, petA, petB, petD, petG, petL, petM, petN, psaA, psaB, psaC, psaD, psaF, psaJ, psaL, psbB, psbC, psbD, psbE, psbF, psbH, psbI, psbJ, psbK, psbL, psbN, psbT, psbV, psbW, psbX, psbY, rbcL, rbcS, rpl1,rpl2,rpl3,rpl4,rpl5,rpl6,rpl11, rpl12, rpl13, rpl14, rpl16, rpl18, rpl19, rpl20, rpl23, rpl24,rpl29,rpl31,rpl34,rpl35, rpoA, rpoB, rpoC1, rpoC2, rps2, rps3, rps4, rps5, rps7, rps8, rps9, rps10, rps11, rps12, rps13, rps14, rps16, rps17, rps18, rps19, rps20, secA, secY, tatC, ycf3, ycf4, ycf39*, and *ycf41*. These genes were analyzed in the PhyloSuite (Zhang et al., 2020), including alignment by Mafft, concatenation by Concatenate Sequence, finding the best-fit evolutionary model by PartitionFinder2 and phylogenetic trees construction using MrBayes methods. Molecular dating was performed using the PAML package (Yang, 2007). Two calibration points were used in the analysis (Supplementary Table 2), including *Synedra* and *Fragilaria* (Nakov et al., 2018), *Ectocarpus siliculosus* and Bacillariophyta (Armbrust, 2009). The phylogenetic tree was visualized in the FigTree with 95% highest posterior density interval (HPD).

### Gene Structure and Phylogenetic Analysis of *petF* Gene Among *Skeletonema* Species

To obtain scaffolds, clean data were assembled independently using Platanus (Kajitani et al., 2019), SPAdes (Bankevich et al., 2012), and ABySS (Jackman et al., 2017). To find *petF* genes encoded in the nuclear genome, sequences of *petF* of published *Skeletonema* species (Roy et al., 2020) were used as references to find the target scaffolds using BLAST (tblastn). Because some software failed to assemble the target scaffolds or the assembly was too fragmented, we combined and compared results of tree assembly software. The target scaffolds were further annotated using genewise^4^ (GeneWise, 2008). Phylogenetic analysis of *petF* genes from cpDNAs and nuclear genomes of *Skeletonema* species were analyzed based on peptide sequences using the Maximum Likelihood (ML) method with 1000 bootstrap replicates in megaX (Kumar et al., 2018). The best-fit model was WAG + G selected by Models. *petF* gene structures were visualized using Gene Structure Display Server (Hu et al., 2015). Four *Thalassiosira* species were added for comparison: The *petF* genes of *T. pseudonana* (EF067921) and *T. weissflogii* (KJ958485) were encoded in cpDNA, and the *petF* genes of *T. oceanica* (EJK54785) and *T. rotula* (MMETSP0913) were encoded by the nuclear genomes (Roy et al., 2020).

## Results

### General Characteristics of Chloroplast Genomes

In this study, we constructed complete cpDNAs of six *Skeletonema* strains (CNS00100, CNS00166, CNS00243, CNS00303, CNS00342, and CNS00438) isolated from Chinese coastal seawaters. These six *Skeletonema* strains were annotated as *S. marinoi*, *S. tropicum*, *S. costatum*, *S. costatum*, *S. pseudocostatum*, and *S. grevillei*, respectively, based on their morphological features and similarity to corresponding reference molecular markers (Liu et al., 2021). Although CNS00243 and CNS00303 were identified as different *Skeletonema* species originally, both the strains were proved to be *S. costatum* by our recent study (Liu et al., 2021). By now, the cpDNA of only a single *Skeletonema* species, which is *S. pseudocostatum*, has been constructed (Hamedi et al., 2019). The cpDNAs of all *Skeletonema* species analyzed in this study showed the typical quadripartite structure in which LSC and SSC regions were separated by a pair of inverted repeats (IRA and IRB) (Figure 1). The sizes of these cpDNAs were very similar, ranging from 126,883 bp (*S. grevillei*) to 127,353 bp (*S. costatum* of CNS303 strain) (Table 1). The sizes of LSCs varied from 63,849 bp (*S. grevillei*) to 64,143 bp (*S. costatum* of both CNS00243 and CNS00303 strains), the sizes of SSCs varied from 26,508 bp (*S*. *pseudocostatum*) to 26,707 bp (*S. costatum* of CNS303 strain), and the sizes of IRs varied from 18,210 bp (*S. grevillei*) to 18,252 bp (*S. costatum* of both CNS00243 and CNS00303 strains). The AT contents of these cpDNAs were also very similar, ranging from 68.42 to 69.29% (Table 1).

**TABLE 1:** The Summary of complete cpDNA feature of the *Skeletonema* species.

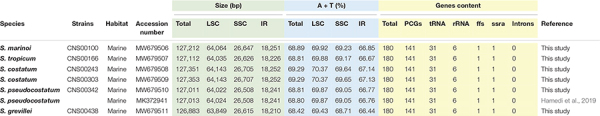

*The strains in blue color were isolated in this study.*

**FIGURE 2:**
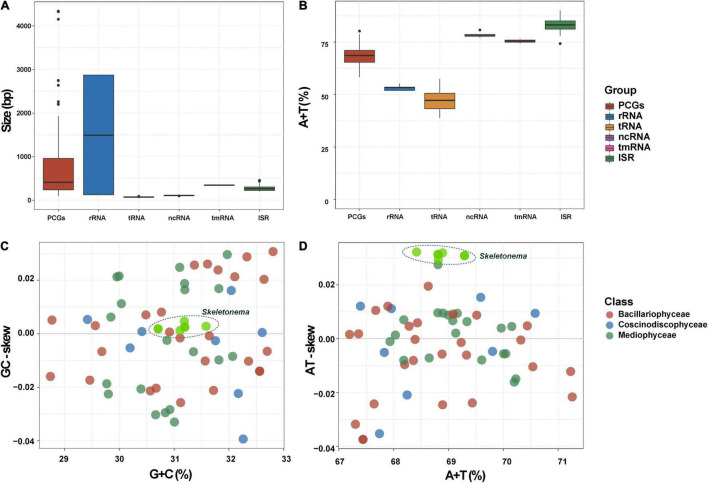
General features of the *Skeletonema* cpDNAs. **(A)** Sizes and **(B)** AT contents of PCG, rRNA, tRNA, ncRNA, tmRNA, and intergenic spacer region (ISR) for the six *Skeletonema* cpDNAs. **(C)** Relationships between G + C contents and GC skews, **(D)** Relationships between A + T contents and AT skews of *Skeletonema* cpDNAs and other 55 published Bacillariophyta cpDNAs.

Consistent with the high similarity of genome sizes and AT contents of these *Skeletonema* species, their gene contents were identical, including 141 PCGs, 31 tRNAs, six rRNAs, one non-coding RNA (ncRNA), and one transfer-messenger RNA (tmRNA) (Table 1). No introns were identified in these six cpDNAs. As expected, the combined lengths corresponding to PCGs were the largest, followed by that corresponding to rRNAs, intergenic spacer regions (ISRs), tRNAs, tmRNAs and ncRNAs (Supplementary Table 3). The sizes of 141 PCGs were rather different, ranging from 90 bp (*petN*) to 4338 bp (*rpoC2*) (Figure 2A). The AT contents did not show much differences among these categories (Figure 2B). The nucleotide skew analysis showed that all the *Skeletonema* cpDNAs exhibited a moderate positive GC-skew (0.0012–0.0047) and a more prominent AT-skew (0.029–0.032) (Figures 2C,D).

### Synteny Analysis and Nucleotide Diversity Analysis

**FIGURE 3:**
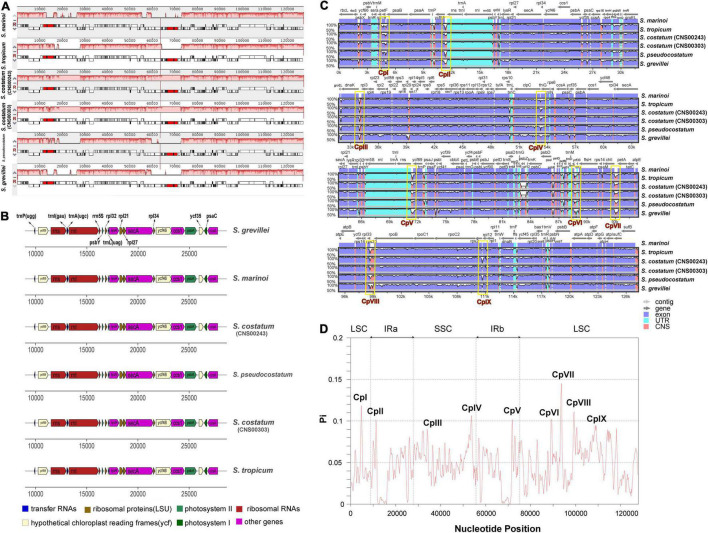
Gene order comparison and the sequence divergence analysis of six *Skeletonema* chloroplast genomes. **(A)** Synteny comparison of the six *Skeletonema* cpDNAs using Mauve analysis. Rectangular blocks of the same color indicate collinear regions. **(B)** Gene arrangements in the inverted repeats (IR) regions of *Skeletonema* cpDNAs. The colors of genes are same with that in the Figure 1, indicating the different functional groups. **(C)** Identity plot comparing the cpDNA of six *Skeletonma* strains using mVISTA. The *S. marinoi* cpDNA (MW679506) is selected as the reference sequence. The vertical scale on the left indicated the identity percentages (range shown: 50–100%). The arrow with light gray named contig on the horizontal axis indicate the cpDNAs of *Skeletonema*. The dark gray arrows above the alignments indicate gene orientation. Genome regions are colored as exon, untranslated region (UTR) and conserved non-coding sequences (CNS). **(D)** Nucleotide diversity (Pi) of the *Skeletonema* cpDNA sequences based on sliding window analysis. The window length is 600 bp and the step size is 200 bp. The horizontal axis indicate the position of the midpoint of a window. The vertical axis indicate the nucleotide diversity of each window. The nine regions with great sequence divergence are also shown in the **(C,D)**, named CpI, CpII, to CpIX.

Alignment of six *Skeletonema* cpDNAs using Mauve revealed highly similar gene arrangements (Figure 3A). No expansion or contraction of IR was observed among these *Skeletonema* cpDNAs (Figure 3B). Sequence identity analysis by mVISTA (Figure 3C) also revealed high similarity across the cpDNAs, with higher conservation observed for PCGs than non-coding regions. The nucleotide diversity exhibited different patterns in the quadripartite structures (Figure 3D), with higher conservation of the IR regions than the LSC and SSC regions.

**FIGURE 4:**
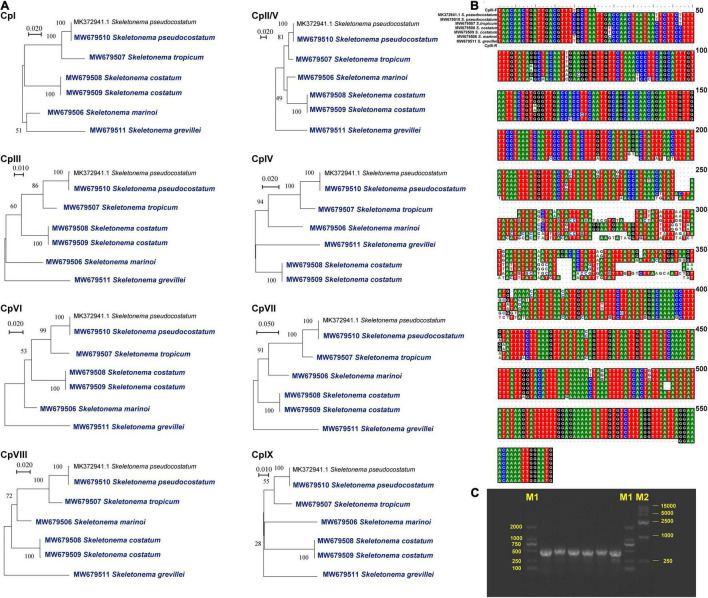
Nine suitable regions were suitable as barcodes or molecular markers. **(A)** Phylogenetic analysis of the nine regions among *Skeletonema* species. DNA alignment information **(B)** and agarose gels image of PCR products **(C)** for CpIII region among the *Skeletonema* species. The bands sequences are *S. marinoi*, *S. tropicum*, *S. costatum* (CNS00243 strain), *S. costatum* (CNS00303 strain), *S. pseudocostatum*, and *S. grevillea*, respectively. The bands of Marker M1 and M2 are shown on both sides. The sequence of CpII region is equal to the CpV region.

Despite the well-conserved genomic structure, we identified nine regions with enhanced variations based on the sequence divergence analysis (Figures 3C,D). These regions, which were named CpI, CpII, CpIII, CpIV, CpV, CpVI, CpVII, CpVIII and CpIX, located in *rpl19 - petF- psaB*, *ycf89 - rns* (IRA), *dnaK - rpl3, clpC - thiG - trnN, rns - ycf89* (IRB), *ycf33 - trnT, chlI - petA, rpl33 - rps20 - rpoB, rpl12 - rpl1*, respectively. The sequences of CpII and CpV regions are identical because they are symmetrically arranged in the IR. To evaluate the divergence of the nine regions, we carried out phylogenetic analysis of these DNA sequences, which showed that these nine regions could all successfully separate all five *Skeletonema* species (Figure 4A). Thus, all of these divergent regions can be used as specific molecular markers for distinguishing different *Skeletonema* species (Figures 4B,C). To test this idea, we have designed primers for amplifying the CpIII region and have successfully amplified this region in all strains (Supplementary Figure 1 and Table 1).

### Phylogenetic Analysis of *Skeletonema* Species

**FIGURE 5:**
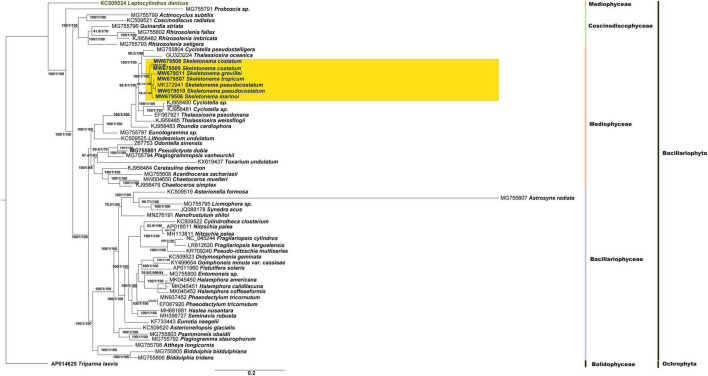
Phylogenetic tree based on concatenated amino acid sequences of 86 chloroplast PCGs. The six *Skeletonema* strains (in blue color) and other 55 Bacillariophyta species, which have been reported the cpDNAs, are included. *Triparma laevis* is used as outgroup taxa.

To explore the evolutionary relationships of *Skeletonema* among the Bacillariophyta species, the phylogenetic tree was constructed using a set of 86 chloroplast PCGs shared by 62 cpDNAs (Figure 5). The phylogenetic tree showed the species in Bacillariophyta were divided into three distinct taxa, which generally corresponded to three classes Bacillariophyceae, Mediophyceae and Coscinodiscophyceae with one exception. *Leptocylindrus danicus* split from other species at the base of the phylogenetic tree and clustered with Coscinodiscophyceae species, suggesting that it was a member of the class Coscinodiscophyceae (Figure 5). This annotation of *L. danicus* was consistent with the previous study (Yu et al., 2018), but was inconsistent with Algaebase (Guiry and Guiry, 2021), which indicated that this species belonged to the class Mediophyceae. *Astrosyne radiata* (MG755807) belonging to Bacillariophyceae was found to have the longest branch, which was consistent with the previous study (Yu et al., 2018) and also reported *A. radiata* exhibited a high level of gene order rearrangement.

As expected, *Skeletonema* species clustered together and formed a monophyletic group close to *Thalassiosira* species. Within *Skeletonema*, the two *S. costatum* strains (MW679508 and MW679509) formed an independent clade, which was sister to the rest of the *Skeletonema* species with 100% bootstrap support. The two *S. pseudocostatum* strains (MW679510 and MK372942), which were isolated from China and Algeria, respectively, clustered together as expected. The clade of *S. pseudocostatum* and *S. tropicum* clustered with the *S. marinoi* (MW679506). The clade including *S. pseudocostatum*, *S. tropicum* and *S. marinoi* was a sister to the clade with a single species: *S. grevillei* (MW679511).

### Selection Analysis and SSR Analysis of the *Skeletonema* cpDNAs

The pairwise substitution rates (Ka/Ks) between the *Skeletonema* species were calculated using PCGs of cpDNA. In this study, the Ka/Ks ratio for all PCGs of all pairwise species comparison was below 1 (Supplementary Figure 2), suggesting purifying selection. Interestingly, the *rbcL* and *rbcS* genes encoding to RubisCO subunit showed higher Ka/Ks ratio than genes belonging to other functional groups.

The distributions of SSRs among *Skeletonema* cpDNAs were analyzed using MISA (Supplementary Table 4). The lengths of SSRs ranged from 10 bp to 22 bp. Most SSRs are A/T type rather than the G/C type. Among the six detected categories, the most abundant were tetra-nucleotide repeats for all *Skeletonema* cpDNAs, accounting for 33.33% (*S. marinoi*)–41.38% (*S. costatum*). The penta-nucleotide repeat was only found in the cpDNAs of *S. costatum*. The number of SSRs located in the ISRs (52.17–63.89%) was slightly higher than that located within the genes (36.11–47.83%). Among the quadripartite structure of *Skeletonema* cpDNAs, SSRs were mainly located in the LSC regions (47.83–53.33%). We found that there was one more SSR (i.e., tetra-nucleotide repeat) in the *S. pseudocostatum* cpDNA (MW679510) of the Chinese strain than that in *S. pseudocostatum* cpDNA (MK372941) of the Algerian strain.

### Divergence Time Estimation Based on the cpDNAs

**FIGURE 6:**
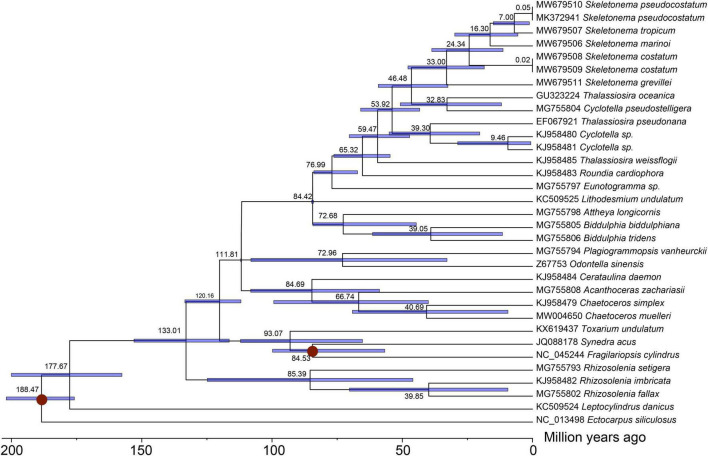
Time-calibrated phylogeny of 33 diatom taxa based on 96 chloroplast genes with PAML. The red dots represented calibration point and translucent blue bars represented the 95% highest posterior density interval of each node ages.

The divergence times of *Skeletonema* species was estimated based on 96 PCGs shared by cpDNAs of 33 species (Figure 6). The divergence time of the *Skeletonema* genus, which formed an independent clade apart from the *Thalassiosira* genus, was estimated to be 46 Million years ago (Mya; 95% HPD: 32.50–59.23 Mya). Within the *Skeletonema* genus, *S. grevillei* diverged from other *Skeletonema* species around 33 Mya (95% HPD: 18.54–47.83 Mya). The two HAB species *S. marinoi* and *S. pseudocostatum* diverged approximately 16 Mya (95% HPD 5.66–29.85 Mya).

### Co-existence of Two Copies of *petF* in *Skeletonema* Species

**FIGURE 7:**
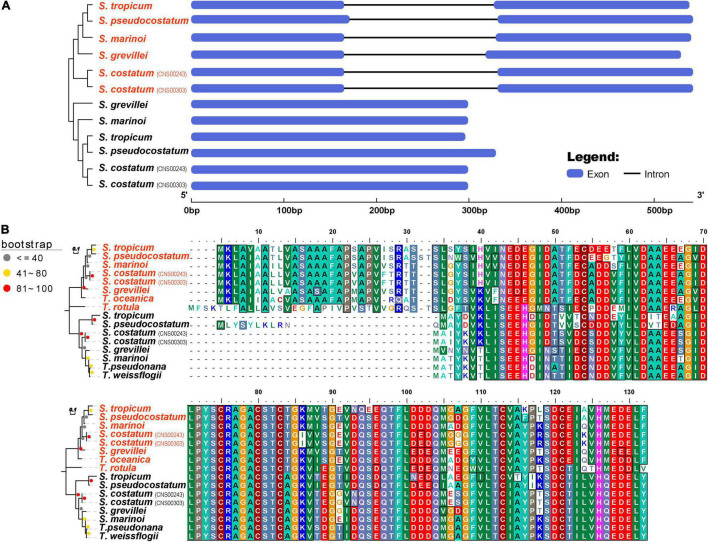
The characteristics of *petF* gene among *Skeletonema* species. **(A)** The phylogenetic analysis and genetic structure and **(B)** the alignment of amino acid sequences. Four *Thalassiosira* species are added for comparison. The *petF* genes of *Skeletonema* strains in red are identified from nuclear genome and these in black are identified from cpDNAs.

The cpDNA gene *petF* has been frequently found to be transferred from cpDNA to nuclear genomes in diatom species (Lommer et al., 2010; Roy et al., 2020). The transfer of *petF* has been proposed as a molecular mechanism underlying iron intake efficacy (Strzepek and Harrison, 2004). A copy of the *petF* gene was found in each of the *Skeletonema* cpDNA (Figure 7A). However, gene annotation of assembled nuclear genomes of all five *Skeletonema* species revealed that the *petF* genes were also found in all of these *Skeletonema* nuclear genomes (Figure 7A), suggesting that *petF* gene transfer did occur in evolution. A typical signal peptide was found at the N-terminal of each nuclear *petF*-encoded peptide, suggesting their role in chloroplast (Figure 7B). Phylogenetic analysis of *petF* genes found in the cpDNAs and nuclear genomes uncovered that all cpDNA-encoded *petF* genes clustered together in one clade (including *T. pseudonana* and *T. weissflogii*), while nuclear *petF* gene clustered together in another clade (including *T. oceanica* and *T. rotula*) (Figure 7B), suggesting that the transfer of *petF* gene occurred before the speciation of these *Skeletonema* species.

## Discussion

*Skeletonema* species have been attracting extensive attention because they frequently dominate the phytoplankton composition along the coastal regions and play important role in primary production. Members of *Skeletonema* species, such as *S. marinoi*, *S. costatum* and *S. pseudocostatum*, can cause blooms with negative impact on environment. While the cpDNA sequences have been proven to be informative for genetic diversity evaluation, cpDNA of only a single species *S*. *pseudocostatum* has been constructed.

### Conserved cpDNAs and Sequence Divergence Analysis Among *Skeletonema* Species

In this study, we reported cpDNAs of five *Skeletonema* species, *S. marinoi, S. tropicum*, *S. costatum* (with two strains), *S*. *pseudocostatum* and *S. grevillei*, increasing the number of cpDNAs of *Skeletonema* species from one to five. These cpDNAs showed remarkable similarities in sizes (Table 1), gene composition, and gene organization (Figures 1, 3). In contrast, cpDNAs of other intra-genus species belonging to the Bacillariophyceae class showed great difference. For example, the cpDNAs of three *Halamphora* species were not conserved, i.e., cpDNAs size (difference up to 28,811 bp), gene order (inversion and translocation), and gene contents (presence/absence of *serC* and *tryC*) (Hamsher et al., 2019).

Among the previously reported cpDNAs of 55 Bacillariophyta species, the sizes varied greatly, ranging from 111,539 bp (KR709240, *Pseudo-nitzschia multiseries*) to 201,816 bp (MG755792, *Plagiogramma staurophorum*). Comparative analysis of these cpDNAs revealed that the main factors contributing to variation of cpDNAs sizes included expansion or contraction of IR regions, gene loss or gain, presence or absence of introns and the length of ISR (Sabir et al., 2014; Yu et al., 2018). The largest cpDNA size of *P. staurophorum* so far discovered was mainly due to the expansion of IR regions (34,888 bp) and a large intron (2971 bp) in *petD* gene (Yu et al., 2018). During evolution, the IR regions have expanded and contracted under various stress condition. It was found that the large sizes of cpDNAs in the order of Thalassiosirales species were mainly contributed by the large size of IR regions (Sabir et al., 2014). The sizes of *Skeletonema* cpDNAs were above average among the reported Bacillariophyta cpDNAs and the lengths of IR regions were above 18,000 bp, which were congruent with the previous study (Sabir et al., 2014).

Loss of genes in the Thalassiosirales order including *petF, petJ, acpP1, ilvB, ilvH, tsf, syfB, serC, syfB*, and *tyrC* from cpDNAs had been previously reported (Ruck et al., 2014; Sabir et al., 2014; Yu et al., 2018). Analysis of cpDNAs of the five *Skeletonema* species confirmed that all of these genes except *petF* were lost from the cpDNAs of these five *Skeletonema* species. The *petF* gene was identified in the cpDNA of all five *Skeletonema* species. The loss of *petF* was found to be caused by the transfer of this gene from chloroplast to nucleus in many species including *T. oceanica*, which may play a role in iron utilization (Lommer et al., 2010; Roy et al., 2020). Interestingly, copies of the *petF* genes were also identified in the nuclear genomes of these five *Skeletonema* species (Figure 7), indicating that each of these five *Skeletonema* species harbor at least two copies of *petF*. The nuclear-encoded *petF* genes of five *Skeletonema* species clustered together in the phylogenetic tree and had one intron in each gene structure (Figure 7), suggesting that *petF* gene transfer from cpDNA to nuclear genome took place before the diversification of these *Skeletonema* species, and intron in the nuclear-encoded *petF* gene had already formed in their common ancestor.

Despite of the high synteny and conserved structure among *Skeletonema* cpDNAs, nine regions with enhanced divergences were detected. These regions with enhanced divergences were proved to be suitable as molecular markers for distinguishing *Skeletonema* species.

Simple sequence repeats can be highly variable at the intra-specific level and were often used as genetic markers in the population genetics studies (Kaur et al., 2015). The SSRs in cpDNAs of the seven *Skeletonema* taxa (including *S. pseudocostatum* of Algerian strain) represented abundant variation, especially for the same species (*S. pseudocostatum*) isolated from different seawaters. Thus, they are useful for detecting genetic polymorphisms among *Skeletonema* species.

### Phylogeny and Selection Patterns Within *Skeletonema* cpDNAs

Phylogenetic tree based on the concatenated amino acid sequences of 86 shared cpDNA PCGs of 62 species showed that *Skeletonema* species formed an independent clade (Figure 5). The phylogenetic analysis supported that *S. pseudocostatum* and *S. tropicum* could clustered into sister clade in agreement with the topology in recent studies using 18S rDNA, 28S rDNA, and *cox1* gens (Kooistra et al., 2008; Yamada et al., 2017). However, the topologies of the phylogenetic trees were not identical, the tree using the cpDNA PCGs obtained in this study was very similar with the tree based on 28S rDNA (Kooistra et al., 2008; Yamada et al., 2017), and was almost identical with the *cox1* tree (except the positions of *S. costatum* and *S. grevillei*) (Liu et al., 2021), but was different from the tree based on 18S rDNA (Yamada et al., 2017), confirming that these different genes can have incongruent histories.

Pairwise substitution rate (Ka/Ks) between cpDNAs of *Skeletonema* species was below 1, indicating all the PCGs of cpDNAs were under purifying selection. The purifying selection was also found in similar analysis performed in other species, such as *Dracunculus* (Abdullah et al., 2021) and *Lagerstroemia* (Xu et al., 2017).

### *Skeletonema* Species Divergence Time Estimation Based on cpDNAs

The *Skeletonema* species were nested within the paraphyletic genus *Thalassiosira* as supported by previous studies (Sorhannus, 2007; Alverson, 2014; Roy et al., 2020). The *Skeletonema* species diverged from *Thalassiosira oceanica* (the nearest species in Figure 6) approximately 46 Mya in the Eocene period, which was slightly later than that estimated using two nuclear genes and two plastid genes (about 55 Mya) (Alverson, 2014). The two HAB species *S. marinoi* and *S. pseudocostatum* diverged approximated 16 Mya (95% HPD 5.66–29.85 Mya), which was consistent with results estimated previously using 18S rDNA (about 14 Mya) (Sorhannus, 2007). The divergence time estimated in this study among *Skeletonema* species was generally similar or slight large than that estimated based on the mtDNA PCGs, indicating the clock rate was similar across the two organelles (Liu et al., 2021). This study provided the divergence time among the *Skeletonema* species based on the cpDNAs for the first time.

## Conclusion

In this study, we constructed complete cpDNAs for five *Skeletonema* species (two strains for *S. costatum*), including cpDNAs of four *Skeletonema* species for the first time. Comparative analysis of the cpDNA sizes, structure, gene number and gene arrangement showed high similarity among *Skeletonema* species. Variations of the IR regions among *Skeletonema* cpDNAs was low, suggesting the IR regions were highly conserved for cpDNAs of *Skeletonema* species, compared with those of other genera, such as the genus *Thalassiosira*. Nine regions with enhanced sequence divergence were obtained, which could be used to distinguish *Skeletonema* species. The PCGs of cpDNAs exhibited depressed Ka/Ks ratios, suggesting that their genes had experienced strong purifying selection to eliminate deleterious mutations. Further information from the whole genome data of *Skeletonema* species is needed to explore their ecological adaptability of different seawaters.

## Data Availability Statement

The datasets presented in this study can be found in online repositories. The names of the repository/repositories and accession number(s) can be found below: The sequencing results (raw data) have been submitted to NCBI and the BioProject number is PRJNA695365. The cpDNAs of six *Skeletonema* strains have been submitted to NCBI and the accession numbers were MW679506–MW679511.

## Author Contributions

NC conceived of the study. YZ helped the sampling from the Jiaozhou Bay. SL, KL, and QX performed analysis. SL and NC interpreted the data and wrote the manuscript. All authors contributed to the editing and gave final approval for publication.

## Conflict of Interest

The authors declare that the research was conducted in the absence of any commercial or financial relationships that could be construed as a potential conflict of interest.

## Publisher’s Note

All claims expressed in this article are solely those of the authors and do not necessarily represent those of their affiliated organizations, or those of the publisher, the editors and the reviewers. Any product that may be evaluated in this article, or claim that may be made by its manufacturer, is not guaranteed or endorsed by the publisher.

## Footnotes

1
https://github.com/BFL-lab/Mfannot

2
https://www.ncbi.nlm.nih.gov/orffinder/

3
http://tree.bio.ed.ac.uk/software/figtree/

4
https://www.ebi.ac.uk/Tools/psa/genewise/
