# Supplementary material for: Chloroplast Genomes for Five Skeletonema Species: Comparative and Phylogenetic Analysis
Source: Front Plant Sci. 2021 Dec 13;12:774617. doi: 10.3389/fpls.2021.774617 (PMC8710728; doi:10.3389/fpls.2021.774617)
Supplement: Supplementary file 1 [file Data_Sheet_1.docx]

**Supplementary Table 1 Primers for PCR amplification of the nine regions.**

| **Regions** | **Forward primer (5′ → 3′)** | **Reverse primer (5′ → 3′)** |
| --- | --- | --- |
| CpI | GGGAATAAAGAGCGAGTT | TTCACTTCTCAGTGGGTT |
| CpII | AAAATTGCAGATTCAGTA | CACGTGTTACTCACCCGTCCGCCAC |
| CpIII | TAACAACTGATTGACGTTTTGCTAA | CATTCCAATTTTGTTTCC |
| CpIV | AATAACTGGATTAGTACAAGA | GGTCGTAGGTTCAAGTCCTACCTGG |
| CpVI | CCACTCAGCCATTTCTCC | TTGCTGTCCCATCTCGTT |
| CpIX | CTGATGCTTCTAATAAAGT | AGATAAAAGTGGTGTTGTA |

**Supplementary Table 2 Calibration points used in the divergence time analysis.**

| **Bracket 1** | **Bracket 2** | **Divergence time (Ma)** | **reference** |
| --- | --- | --- | --- |
| *Synedra* | *Fragilaria* | 56-126 | (Nakov, et al. 2018) |
| *Ectocarpus siliculosus* | Diatoms (Bacillariophyta) | 176-202 | (Armbrust 2009) |

Reference

Armbrust EV 2009. The life of diatoms in the world's oceans. Nature 459: 185-192. doi: 10.1038/nature08057

Nakov T, Beaulieu JM, Alverson AJ 2018. Accelerated diversification is related to life history and locomotion in a hyperdiverse lineage of microbial eukaryotes (Diatoms, Bacillariophyta). New Phytol 219: 462-473. doi: 10.1111/nph.15137

**Supplementary Table 3 The combined lengths of PCGs, ISRs, rRNA, tRNA, ncRNA, tmRNA in cpDNA of *Skeletonema* species.**

| Classification | Combined lengths |
| --- | --- |
| PCGs | 588123 |
| ISRs | 37014 |
| tRNA | 14058 |
| tmRNA | 2056 |
| ncRNA | 644 |

**Supplementary Table 4 Distribution of each SSR category in the seven *Skeletonema* cpDNAs**.

| **Species** | **Category** | **Number** | **Intergenic** | **Gene** | **LSC** | **SSC** | **Ira** | **Irb** |
| --- | --- | --- | --- | --- | --- | --- | --- | --- |
| *S. marinoi* | Mono-nucleotide | 4 | 1 | 3 | 2 | 0 | 1 | 1 |
| MW679506 | Di-nucleotide | 7 | 7 | 0 | 4 | 1 | 1 | 1 |
|  | Tri-nucleotide | 6 | 0 | 6 | 1 | 1 | 2 | 2 |
|  | Tetra-nucleotide | 9 | 7 | 2 | 7 | 0 | 1 | 1 |
|  | Penta-nucleotide | 0 | 0 | 0 | 0 | 0 | 0 | 0 |
|  | hexa-nucleotide | 1 | 0 | 1 | 0 | 1 | 0 | 0 |
|  | Subtotal | 27 | 15 | 12 | 14 | 3 | 5 | 5 |
| *S.tropicum* | Mono-nucleotide | 6 | 2 | 4 | 3 | 1 | 1 | 1 |
| MW679507 | Di-nucleotide | 6 | 6 | 0 | 2 | 2 | 1 | 1 |
|  | Tri-nucleotide | 6 | 0 | 6 | 2 | 0 | 2 | 2 |
|  | Tetra-nucleotide | 11 | 8 | 3 | 9 | 0 | 1 | 1 |
|  | Penta-nucleotide | 0 | 0 | 0 | 0 | 0 | 0 | 0 |
|  | hexa-nucleotide | 1 |  | 1 | 0 | 1 | 0 | 0 |
|  | Subtotal | 30 | 16 | 14 | 16 | 4 | 5 | 5 |
| *S. costatum* | Mono-nucleotide | 5 | 1 | 4 | 3 | 0 | 1 | 1 |
| MW679508 | Di-nucleotide | 5 | 5 | 0 | 2 | 3 | 0 | 0 |
|  | Tri-nucleotide | 4 | 0 | 4 | 1 | 1 | 1 | 1 |
|  | Tetra-nucleotide | 12 | 9 | 3 | 8 | 0 | 2 | 2 |
|  | Penta-nucleotide | 2 | 2 | 0 | 0 | 0 | 1 | 1 |
|  | hexa-nucleotide | 1 | 0 | 1 | 0 | 1 | 0 | 0 |
|  | Subtotal | 29 | 17 | 12 | 14 | 5 | 5 | 5 |
| *S. costatum* | Mono-nucleotide | 5 | 1 | 4 | 3 | 0 | 1 | 1 |
| MW679509 | Di-nucleotide | 5 | 5 | 0 | 2 | 3 | 0 | 0 |
|  | Tri-nucleotide | 4 | 0 | 4 | 1 | 1 | 1 | 1 |
|  | Tetra-nucleotide | 12 | 9 | 3 | 8 | 0 | 2 | 2 |
|  | Penta-nucleotide | 2 | 2 | 0 | 0 | 0 | 1 | 1 |
|  | hexa-nucleotide | 1 | 0 | 1 | 0 | 1 | 0 | 0 |
|  | Subtotal | 29 | 17 | 12 | 14 | 5 | 5 | 5 |
| *S. pseudocostatum* | Mono-nucleotide | 6 | 3 | 3 | 3 | 1 | 1 | 1 |
|  | Di-nucleotide | 7 | 7 | 0 | 4 | 3 | 0 | 0 |
| MW679510 | Tri-nucleotide | 6 | 0 | 6 | 2 | 0 | 2 | 2 |
|  | Tetra-nucleotide | 14 | 11 | 3 | 9 | 1 | 2 | 2 |
|  | Penta-nucleotide | 0 | 0 | 0 | 0 | 0 | 0 | 0 |
|  | hexa-nucleotide | 3 | 2 | 1 | 0 | 1 | 1 | 1 |
|  | Subtotal | 36 | 23 | 13 | 18 | 6 | 6 | 6 |
| *S. pseudocostatum* | Mono-nucleotide | 6 | 3 | 3 | 3 | 1 | 1 | 1 |
|  | Di-nucleotide | 7 | 7 | 0 | 4 | 3 | 0 | 0 |
| MK372941.1 | Tri-nucleotide | 6 | 0 | 6 | 2 | 0 | 2 | 2 |
|  | Tetra-nucleotide | 13 | 10 | 3 | 8 | 1 | 2 | 2 |
|  | Penta-nucleotide | 0 | 0 | 0 | 0 | 0 | 0 | 0 |
|  | hexa-nucleotide | 3 | 2 | 1 | 0 | 1 | 1 | 1 |
|  | Subtotal | 35 | 22 | 13 | 17 | 6 | 6 | 6 |
| *S. grevillei* | Mono-nucleotide | 5 | 2 | 3 | 1 | 0 | 2 | 2 |
| MW679511 | Di-nucleotide | 3 | 3 | 0 | 2 | 1 | 0 | 0 |
|  | Tri-nucleotide | 6 | 1 | 5 | 3 | 1 | 1 | 1 |
|  | Tetra-nucleotide | 8 | 6 | 2 | 5 | 1 | 1 | 1 |
|  | Penta-nucleotide | 0 | 0 | 0 | 0 | 0 | 0 | 0 |
|  | hexa-nucleotide | 1 | 0 | 1 | 0 | 1 | 0 | 0 |
|  | Subtotal | 23 | 12 | 11 | 11 | 4 | 4 | 4 |

**Supplementary Figure 1 The agarose gels image of PCR products for the nine regions.** The sequences of CpII region was equal to the CpV region and located in IRA and IRB, respectively. The full-lengt**h gels are presented in Supplementary Figure 3 and Supplementary Figure 4.**


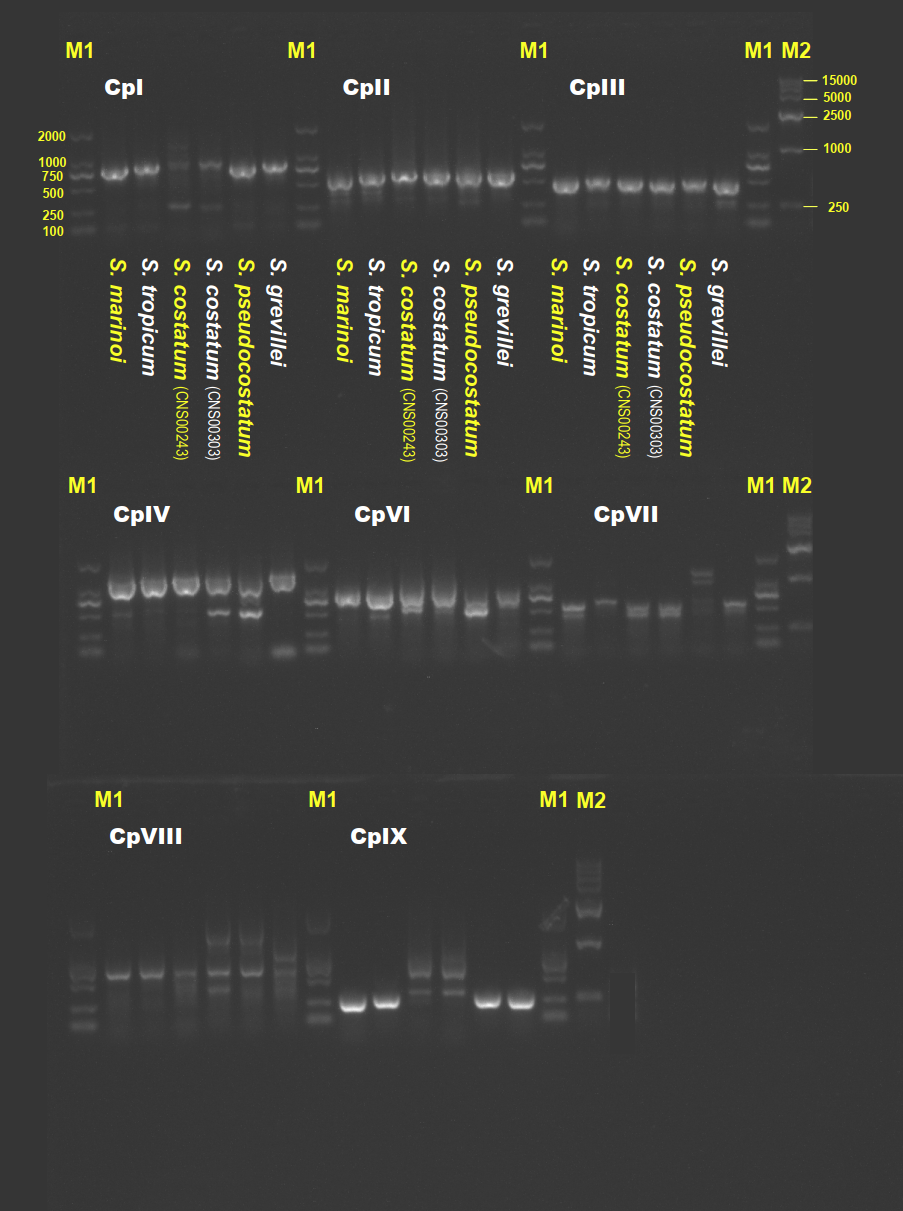


**Supplementary Figure 2 Comparison of Ka/Ks ratios for the chloroplast gene among *Skeletonema* species**.


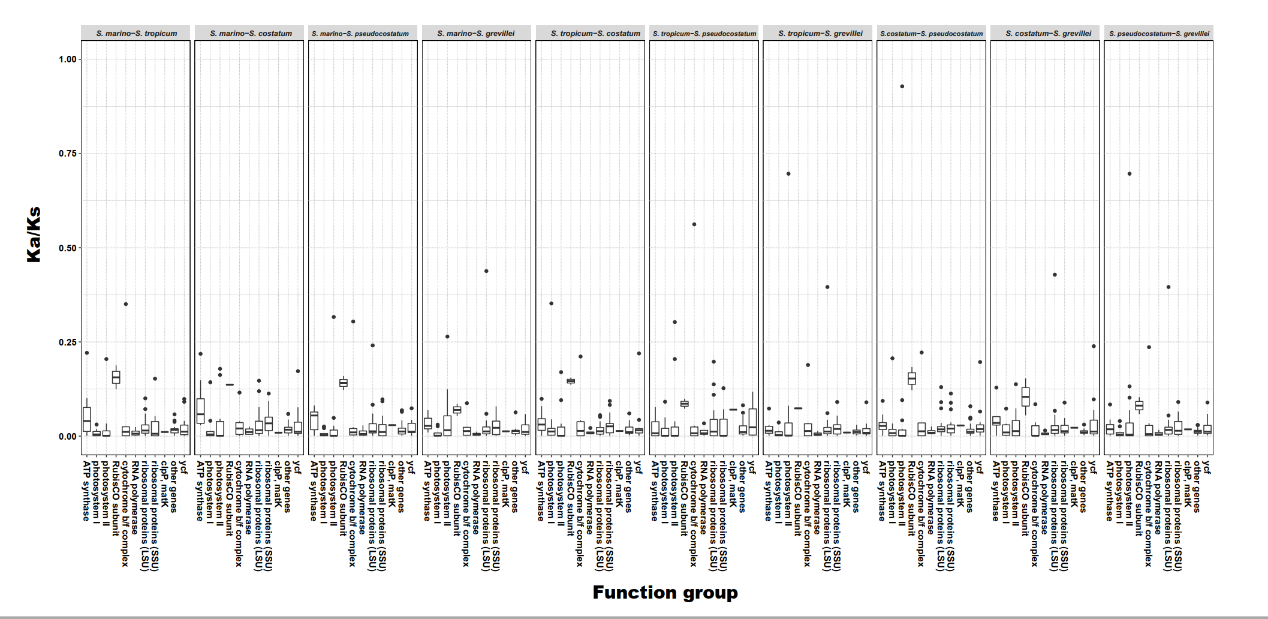


**Supplementary Figure 3** The full-length gels used in **Supplementary Figure 1**.





**Supplementary Figure 4** The full-length gels used in **Supplementary Figure 1**.
